# Tissue acidosis does not mediate the hypoxia selectivity of [^64^Cu][Cu(ATSM)] in the isolated perfused rat heart

**DOI:** 10.1038/s41598-018-36145-1

**Published:** 2019-01-24

**Authors:** Friedrich Baark, Fiona Shaughnessy, Victoria R. Pell, James E. Clark, Thomas R. Eykyn, Philip Blower, Richard Southworth

**Affiliations:** 1 School of Biomedical Engineering & Imaging Sciences, King’s College London, London, UK; 2 School of Cardiovascular Medicine and Sciences, BHF Centre, King’s College London, London, UK

## Abstract

Copper-64-Diacetyl-bis(N^4^-methylthiosemicarbazone) [^64^Cu][Cu(ATSM)] is a hypoxia-targeting PET tracer with applications in oncology and cardiology. Upon entering a hypoxic cell, [^64^Cu][Cu(II)(ATSM)] is reduced to a putative [^64^Cu][Cu(I)(ATSM)]^−^ species which dissociates to deposit radiocopper, thereby providing hypoxic contrast. This process may be dependent upon protonation arising from intracellular acidosis. Since acidosis is a hallmark of ischemic tissue and tumors, the hypoxia specificity of [^64^Cu][Cu(ATSM)] may be confounded by changes in intracellular pH. We have therefore determined the influence of intracellular pH on [^64^Cu][Cu(ATSM)] pharmacokinetics. Using isolated perfused rat hearts, acidosis was induced using an ammonium pre-pulse method, with and without hypoxic buffer perfusion. Cardiac [^64^Cu][Cu(ATSM)] pharmacokinetics were determined using NaI detectors, with intracellular pH and cardiac energetics monitored in parallel by ^31^P NMR. To distinguish direct acidotic effects on tracer pharmacokinetics from acidosis-induced hypocontractility, parallel studies used lidocaine perfusion to abolish cardiac contraction. Hypoxic myocardium trapped [^64^Cu][Cu(ATSM)] despite no evidence of it being acidotic when characterised by ^31^P NMR. Independent induction of tissue acidosis had no direct effect on [^64^Cu][Cu(ATSM)] pharmacokinetics in either normoxic or hypoxic hearts, beyond decreasing cardiac oxygen consumption to alleviate hypoxia and decrease tracer retention, leading us to conclude that tissue acidosis does not mediate the hypoxia selectivity of [^64^Cu][Cu(ATSM)].

## Introduction

Myocardial hypoxia is a major factor in the pathology of cardiac ischaemia, and has been implicated in the progression of numerous events associated with myocardial infarction and heart failure^1–4^, microvascular disease and cardiac hypertrophy^5,6^. Hypoxia is also a well-established characteristic of many cancers, induced by a chaotic vascular architecture which leads to both poor perfusion and decreased oxygen delivery, which frequently combine to limit the effectiveness of chemotherapy and radiotherapy^7^. The non-invasive quantification of tissue hypoxia by molecular imaging is therefore an attractive prospect for disease diagnosis, stratification, and predicting or determining response to therapy in both cancer and cardiovascular disease^8,9^.

**Figure 1:**
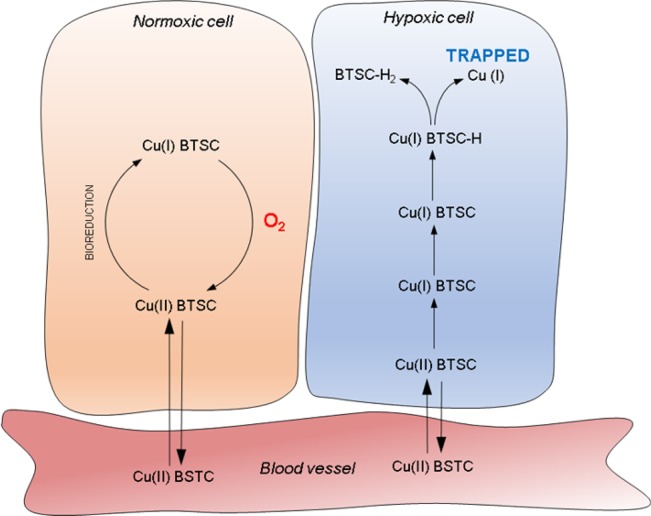
(left) Structure of [Cu(ATSM)], (right) Generalised schematic of the proposed trapping mechanisms for [^64^Cu][Cu(BTSC)] PET tracers. [^64^Cu][Cu(II)(BTSCs)] passively diffuse into cells where they can be reduced to a charged Cu(I) complex which is unable to leave the cell. In the presence of oxygen this Cu(I) complex is rapidly reoxidised back to Cu(II) which is again able to diffuse out of the cell. If oxygen is insufficient, the Cu(I) complex can become further reduced and dissociate. The Cu(I) then becomes sequestered by copper chelating proteins and trapped inside the cell (Adapted with permission from Pell *et al*. 2018 under a CC BY open access licence)^42^.

Radiolabeled Copper-*bis*(thiosemicarbazones) [^64^Cu][Cu(BTSCs)] are a family of hypoxia-avid positron emission tomography (PET) tracers which have been extensively investigated for applications in both oncology and cardiovascular medicine^10–12^. Copper-64-Diacetyl-*bis*(N^4^-methylthiosemicarbazone) [^64^Cu][Cu(ATSM)] is the current lead member of the class, which has been demonstrated to delineate hypoxic tissue in tumors^10,13^ and myocardium^14,15^ pre-clinically and clinically. While their sensitivity to tissue hypoxia is well-established, the exact mechanism of how this class of complexes deliver hypoxia-dependent contrast remains somewhat unclear^16^. As neutral lipophilic molecules, it is thought that they passively diffuse into and out of cells in their oxidized state. Under hypoxic conditions, they are reduced from copper (II) to an unstable copper (I) species^9^ which dissociates, releasing their radiocopper core to become trapped inside the cell, thereby providing hypoxia-dependent imaging contrast^17^ (Fig. 1). While it is not controversial that hypoxia promotes tissue radiocopper retention from these complexes, the intracellular interactions responsible for their reduction remain unidentified^18^. One element which could affect this process either directly or indirectly is intracellular pH. Ligand protonation due to acidosis may alter the redox potential of the oxidised Copper (II) species, thereby altering the lipophilicity of the reduced Copper (I) species, or the dissociation kinetics of the complex^19^. *In vitro* UV-Vis spectroscopy studies have demonstrated that the stability of various [^64^Cu][Cu(BTSCs)], such as PTSM and KTS complexes and their reduction products decrease substantially with reduced pH^11,19^, making them more prone to dissociation, while cyclic voltammetry suggests that [^64^Cu][Cu(ATSM)] is more readily reduced in acidic conditions^19^.

Ischemic myocardium and the tumour microenvironment are both potentially associated with acidosis. In myocardium, the net hydrolysis of ATP during ischemia leads to an increase in intracellular H^+^ concentration^20^, which has been demonstrated by ^31^P NMR spectroscopy to be more severe than that caused by hypoxia alone^21^. Commonly associated with hypoxia, acidosis is also a hallmark of aggressive tumors, driven by a glycolytic phenotype, the increased production and extrusion of H^+^ and lactate, and limited perfusion^22^. The importance of acidosis is of increasing interest as a driver of tumor progression and metastatic spread^22^. If the hypoxia sensitivity of the [^64^Cu][Cu(BTSC)] complexes is mediated by (or heavily modified by) changes in intracellular or intra-tissue pH, this would greatly complicate the use and interpretation of the imaging data that they provide. While *in vitro* studies and *in silico* modeling^19^ suggest that pH may influence the hypoxia selectivity of these complexes, the issue has not yet been specifically investigated in a biologically relevant model of tissue hypoxia.

We have established an isolated perfused heart system coupled with a triple NaI gamma detection apparatus which allows the characterization of radiotracer pharmacokinetics in an intact functioning organ over which we have complete functional control^15,23^. Interventions can be performed accurately and reproducibly without the added complications of circulating tracer metabolites, under conditions which may otherwise be lethal *in vivo*. In this study, we have used this approach to investigate the interplay between tissue hypoxia and intracellular pH, independently and in combination, on radiotracer kinetics to establish whether tissue acidosis is fundamental to, or impacts upon, the hypoxia selectivity of [^64^Cu][Cu(ATSM)].

## Results

### Cardiac hemodynamics

**Figure 2:**
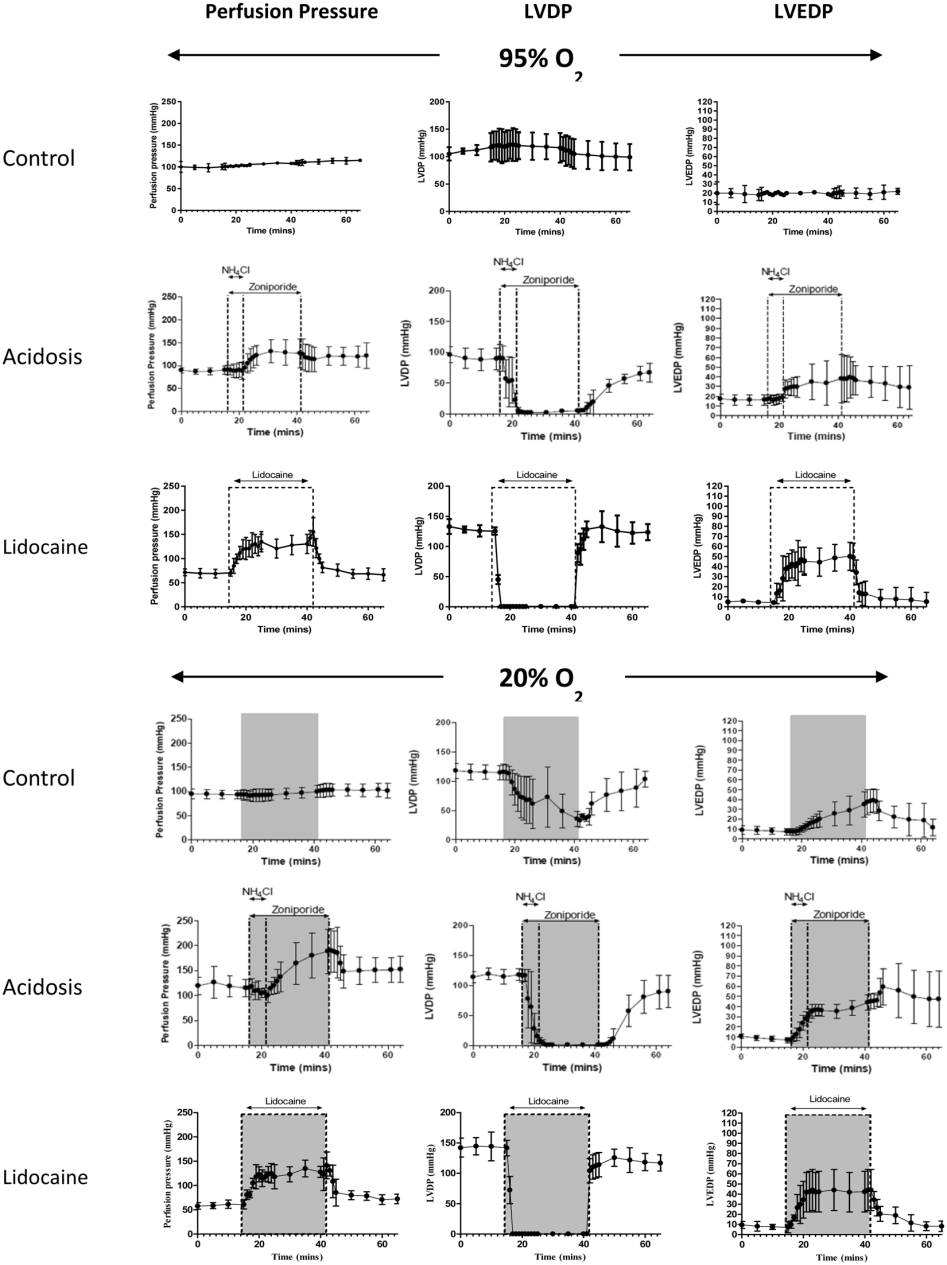
Langendorff hemodynamic data showing changes in perfusion pressure, left ventricular developed pressure and left ventricular end-diastolic pressure from all treatment groups. Data are expressed as mean +/− SD (n = 5).

Cardiac hemodynamic parameters from hearts across all treatment groups are shown in Fig. 2. Hypoxia invoked a rapid decrease in left ventricular developed pressure (LVDP), falling from 115.4 ± 13.4 to 35.5 ± 12.5 mmHg, with left ventricular end-diastolic pressure (LVEDP) increasing from 7.5 ± 3.4 to 35.2 ± 12.9 mm, associated with a slight increase in coronary perfusion pressure (PP) from 93.4 ± 9.2 to 101.7 ± 15.6 mmHg. Ammonium prepulse-induced intracellular acidosis invoked a rapid reduction in LVDP from 90.7 ± 21.9 to 22.5 ± 11.1 mmHg within 5 mins in normoxic hearts, coupled with an increase in LVEDP in from 16.4 ± 5.3 to 38.2 ± 24.8 mmHg after 25 mins, with an increase in PP from 90.5 ± 10.00 mmHg to 131.1 ± 25.2 mmHg. Washout of NH_4_Cl caused a further decrease to LVDP 5.5 ± 4.7 mmHg within 1 minute. After zoniporide washout, LVDP returned to 67.2 ± 15.2 mmHg. The functional effects of acidosis and hypoxia combined were not significantly different from those of acidosis alone, although the complete cessation of cardiac contraction caused by acidosis was more severe than the hypocontractile effect induced by hypoxic buffer perfusion hypoxia alone. Infusion of 0.8 mM lidocaine induced comparable changes in cardiac function to those of the acidosis protocols. In both normoxic and hypoxic hearts, lidocaine abolished LVDP from 125.6 ± 6.1 to 0.54 ± 0.2 and 139.7 ± 7.9 to 0.47 ± 0.13 respectively, returning to 123.7 ± 13.2 and 125.7 ± 14.06 after washout. LVEDP was increased with the addition of lidocaine from 3.5 ± 0.2 to 50.2 ± 14.02 in normoxic hearts and from 6.7 ± 0.68 to 62.36 ± 20.3 in hypoxic hearts. Both of which returned close to pre-infusion values after lidocaine washout.

### ^31^P NMR spectroscopy

**Figure 3:**
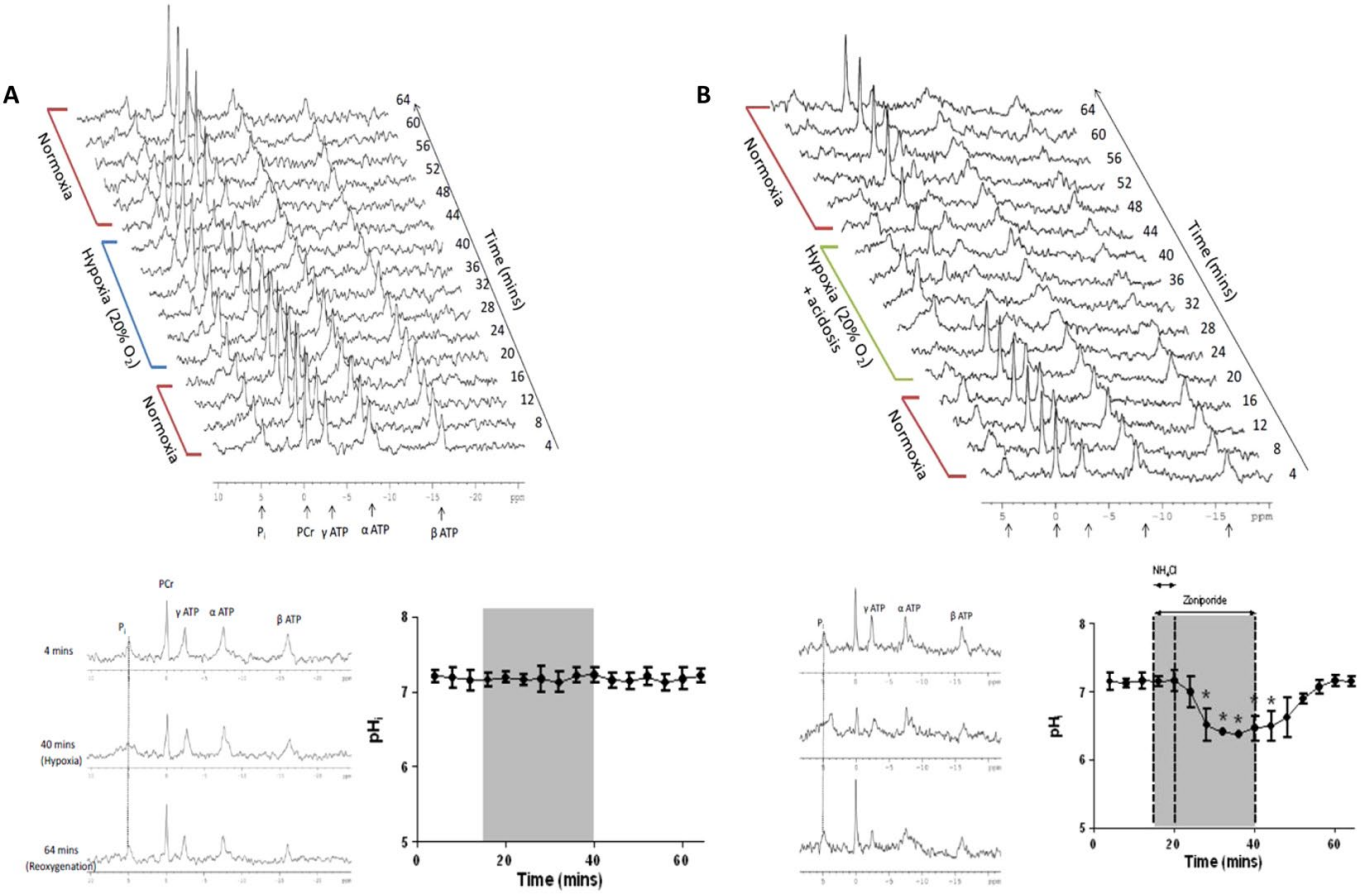
(**A**) Representative stacked plot of ^31^P NMR spectra acquired from a hypoxic control (20% O_2_) heart along with single spectra stacked plot and myocardial pH_i_. **(B)** Representative stacked plot of ^31^P NMR spectra acquired from a hypoxic (20% O_2_) acidotic heart along with single spectra stacked plot and myocardial pH_i_ (data are expressed as mean ± standard deviation, n = 5).

Figure 3 displays representative ^31^P NMR spectra and average pH_i_ from (A) hearts subjected to hypoxia (20% O_2_) and (B) hypoxia + acidosis, to assess changes in cardiac energetics and myocardial pH_i_. Hypoxia with 20% O_2_ did not affect pH_i_, which averaged between 7.13 ± 0.11 and 7.22 ± 0.1 and did not differ from time-matched normoxic control pH_i_. Perfusion with 20% O_2_ KHB plus NH_4_Cl and zoniporide caused pH_i_ to decrease from 7.1 ± 0.07 to 6.4 ± 0.06 (p < 0.05), returning to 7.1 ± 0.10 (which was comparable to time-matched normoxic controls) 15 minutes after zoniporide washout.

### Cardiac lactate release

**Figure 4:**
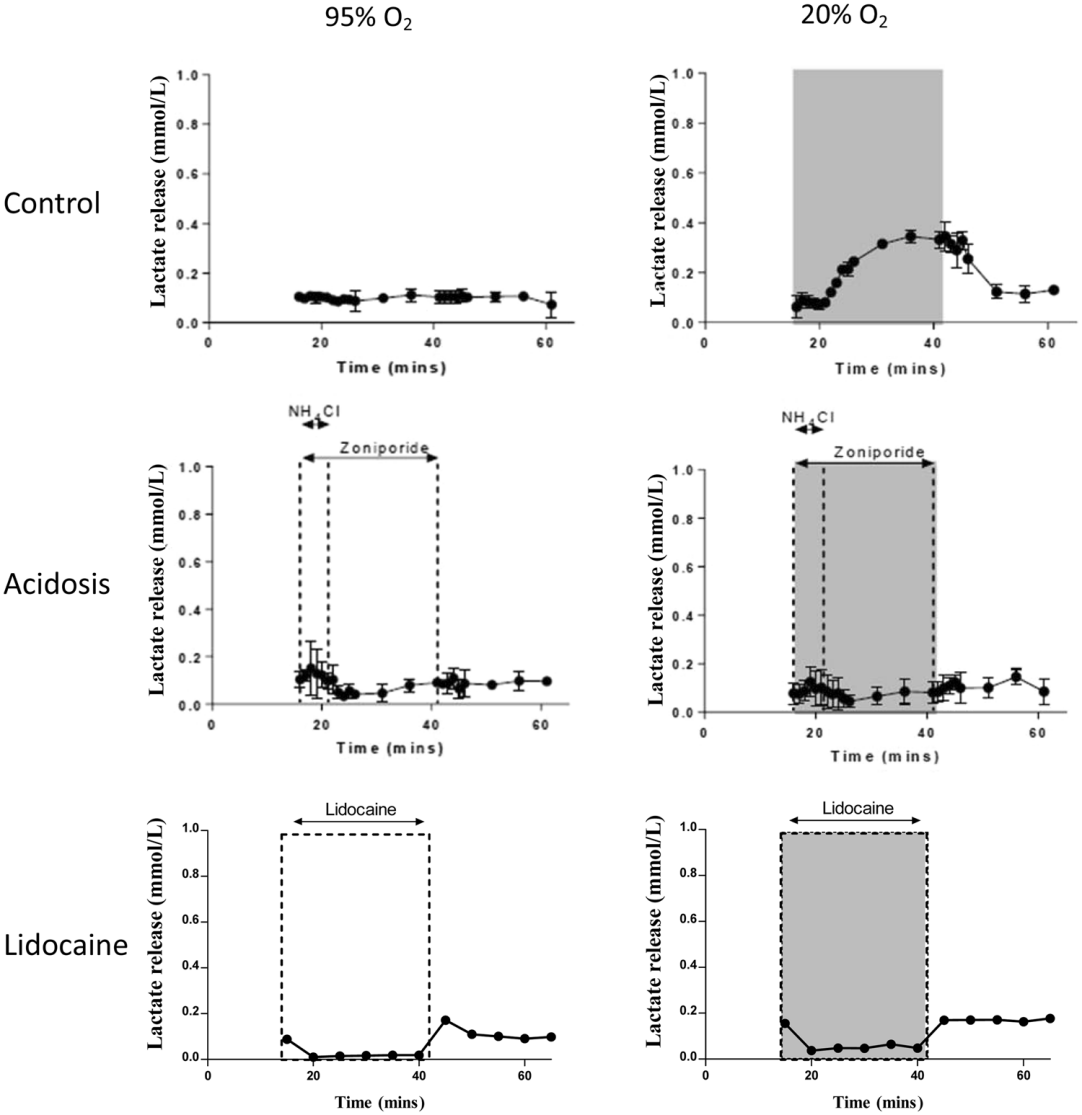
Cardiac lactate release. Lactate concentration was measured in perfusate samples collected from isolated perfused hearts. Data are expressed as means ± SD (n = 5).

Lactate release from hearts in all treatment groups are summarized in Fig. 4. Lactate release from normoxic control hearts was minimal during normoxic perfusion (0.085 ± 0.01 to 0.011 ± 0.03 mmol/L). Perfusion with hypoxic buffer caused elevated lactate washout, reaching a maximum of 0.35 ± 0.03 mmol/L after 20 mins of hypoxia. In normoxic hearts, perfusion with NH_4_Cl caused a transient but not significant small increase in lactate release from 0.10 ± 0.03 to 0.15 ± 0.11 mmol/L within 2 minutes which decreased again upon NH_4_Cl washout. Lactate release from acidotic hearts perfused with hypoxic buffer did not increase throughout the protocol and were not significantly different from non-acidotic control hearts. Lidocaine infusion abolished lactate release during normoxic buffer perfusion, which was not significantly different from the low lactate washout observed in the acidotic group, but significantly lower than that observed in the hypoxia alone perfusion group (p < 0.05).

### Cardiac Copper-64 retention

**Figure 5:**
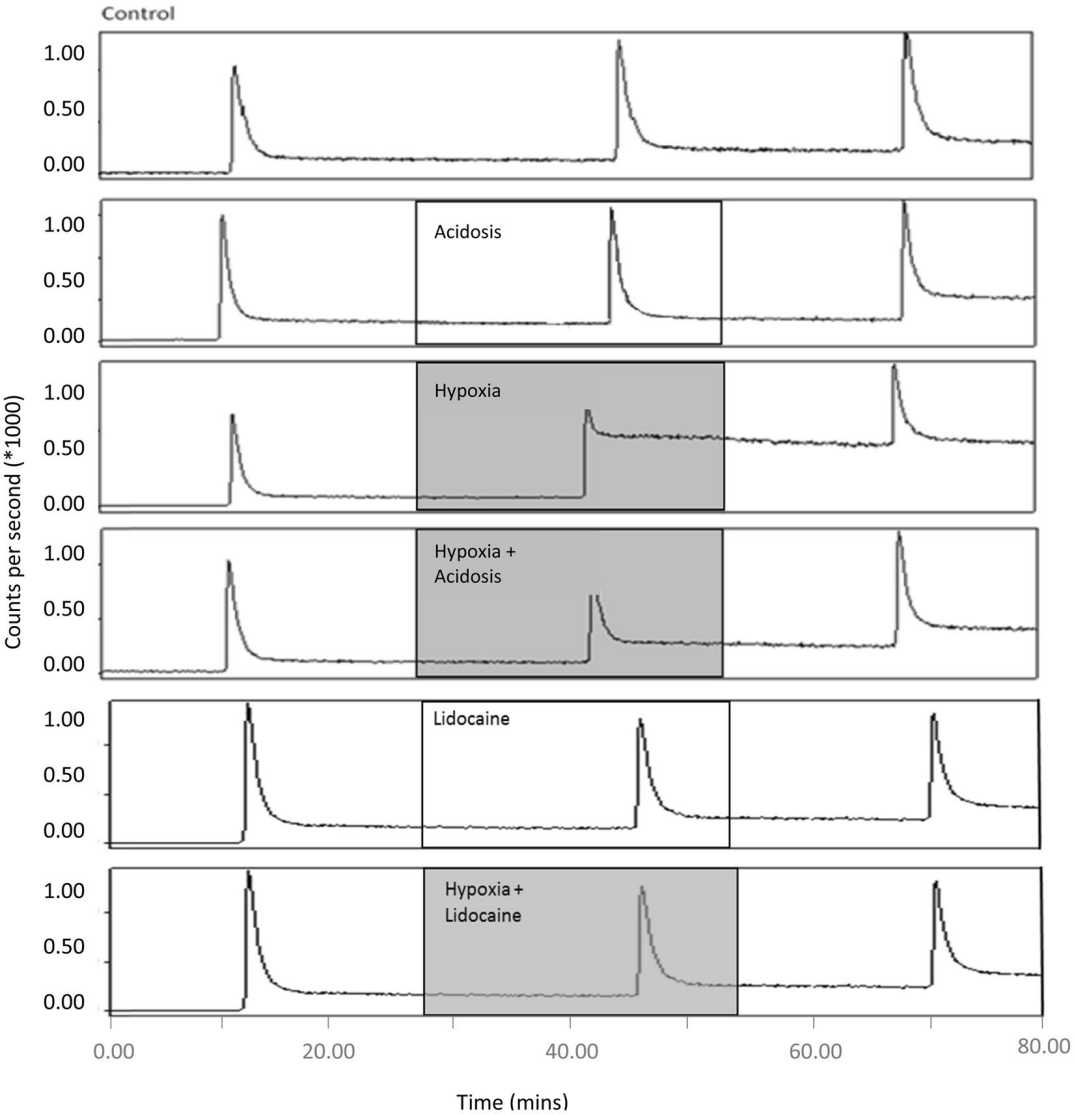
Representative time-activity curves demonstrating Copper-64 retention from [^64^Cu][Cu(ATSM)] across all treatment groups.

**Figure 6:**
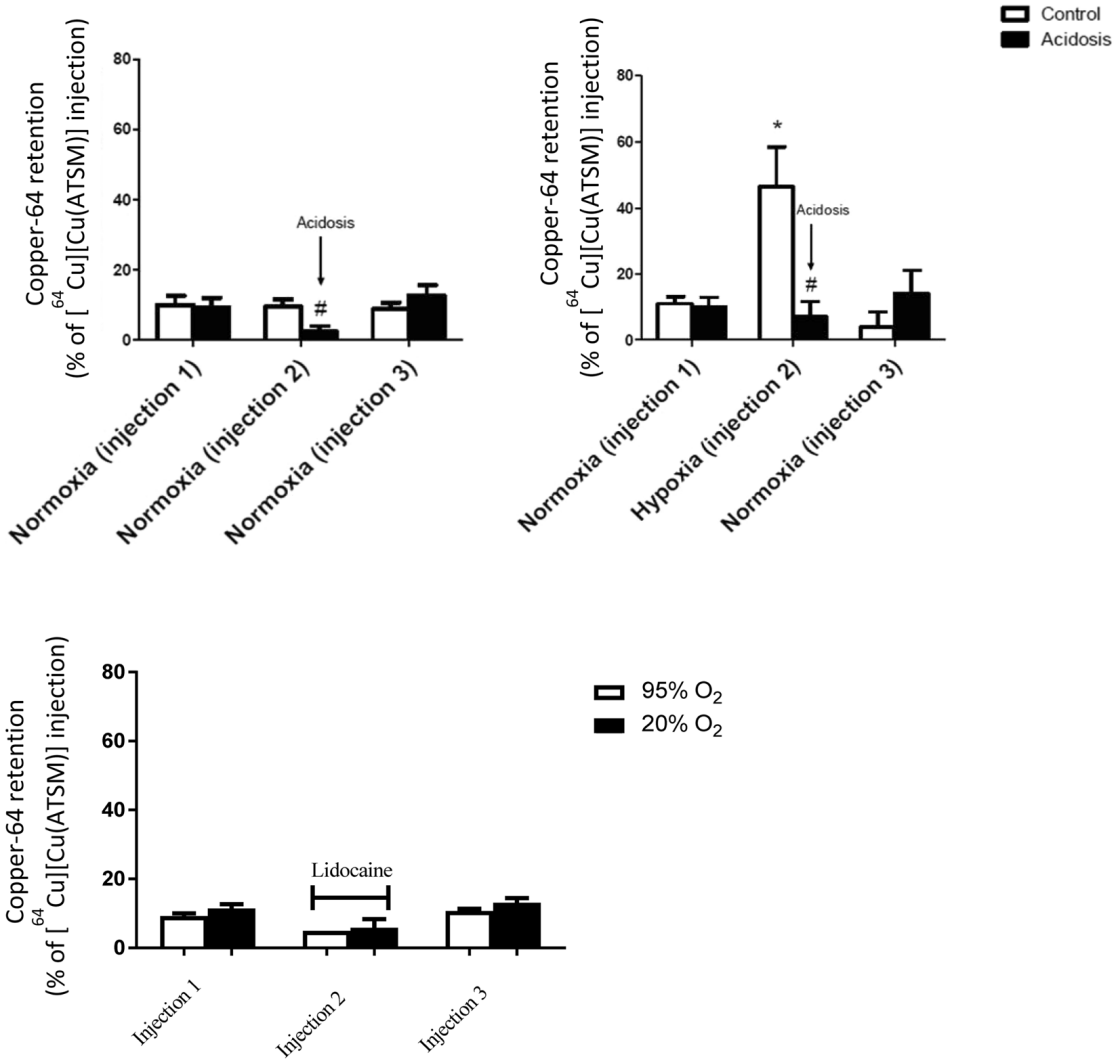
Tissue retention of [^64^Cu][Cu(ATSM)] (% of injected dose) across all treatment groups. Data are expressed as mean ± SD. (n = 5).

Representative traces from the Na/I detector interrogating the heart during examples of each perfusion protocol are shown in Fig. 5, and summarized across all hearts in Fig. 6. During normoxic perfusion, 10.1% ± 2.5% of the injected dose (ID) was retained in the heart 20 min after the first injection, and this level of radiotracer retention was consistently observed in the subsequent injections in the normoxic control heart. Induction of acidosis in normoxic hearts caused a decrease in cardiac Copper-64 retention from 9.3% ± 2.7% during the control period to 2.4% ± 1.6% (p < 0.05), returning to 12.7% ± 3.0% 15 mins after zoniporide/acid washout. Perfusion with hypoxic buffer caused a significant increase in tracer retention from 11.0% ± 2.2% to 46.5% ± 12.0% ID (p < 0.05). However, inducing acidosis in hypoxic hearts abolished Copper-64 retention such that it was not significantly different to pre-hypoxic values. Lidocaine infusion in normoxic hearts had a similar effect to acidosis, with Copper-64 retention falling from 9.22% ± 0.79% to 4.93% ± 0.8% (p < 0.05) before returning to pre-lidocaine values (10.7% ± 0.6% ID). Lidocaine infusion also abolished the elevated Copper-64 retention previously seen in hypoxic hearts.

## Discussion

In this study we show that, contrary to prior suggestions based on *in vitro* physicochemical and electrochemical studies and *in silico* calculations, acidosis is not a significant mechanism for the trapping of [^64^Cu][Cu(ATSM)] in hypoxic tissues *ex vivo*. We demonstrate significant cardiac retention of [^64^Cu][Cu(ATSM)] in myocardium perfused with hypoxic buffer, which exhibits no measurable intracellular acidosis when measured by ^31^P NMR spectroscopy. Acidosis can therefore not be a prime determinant of tissue [^64^Cu][Cu(ATSM)] retention. While acidosis is a well characterized phenomenon in ischemic hearts, and has been robustly demonstrated by ^31^P NMR *ex vivo* and *in vivo* by ourselves and many others^24–26^, maintaining coronary flow constant to specifically induce hypoxia (which our model allows) washes protons arising from net ATP hydrolysis from the myocardium sufficiently rapidly that they do not cause measurable tissue acidosis. Thus, our model allows us to specifically demonstrate the hypoxia-dependent tissue accumulation of [^64^Cu][Cu(ATSM)] without the confounding effects of changes in perfusion which often complicate such studies in cancer models, and to confirm the lack of correlation between acidosis and [^64^Cu][Cu(ATSM)] retention. We show that surprisingly, rather than promoting [^64^Cu][Cu(ATSM)] dissociation and Copper-64 retention as might be predicted, when invoked pharmacologically either in the presence or absence of hypoxia, acidosis indirectly *decreases* [^64^Cu][Cu(ATSM)] retention in our experimental model by lowering cardiac oxygen consumption via the inhibition of cardiac contraction. We mimicked this condition by inhibiting cardiac contraction by lidocaine infusion to achieve the same effect, such that tissues were no longer sufficiently hypoxic to retain [^64^Cu][Cu(ATSM)], despite being perfused with hypoxic buffer. We demonstrate the oxygen-salving effect of this mechanical unloading by the normalization of cardiac energetics (by ^31^P NMR spectroscopy), and the abolition of lactate washout from hypoxic hearts (which reflects a return from anaerobic to aerobic glycolysis) when either simultaneously made acidotic, or perfused with lidocaine. As we have previously demonstrated, [^64^Cu][Cu(ATSM)] exhibits a sigmoidal relationship between tissue oxygenation and tracer retention, and is selective for relatively extreme degrees of hypoxia^27^; by lowering oxygen demand in our hearts, we conclude that we have indirectly raised intracardiac oxygen saturation to above the threshold necessary for tissue [^64^Cu][Cu(ATSM)] retention. This oxygen-sparing effect is also observable in acidotic hearts during normoxic perfusion, where baseline [^64^Cu][Cu(ATSM)] retention was lower than in untreated normoxic control hearts. While the Langendorff isolated perfused heart is energetically stable for several hours, it is potentially at the brink of normoxia because KHB has a lower oxygen-carrying capacity than blood, even when saturated with 95% O_2_/5% CO_2_. This is apparent from the significant vasodilation and high coronary flows associated with crystalloid versus blood perfused preparations^28^. Isolated hearts perfused with KHB are almost maximally vasodilated, which has led to the suggestion that they may be slightly hypoxic, particularly in the endocardium, where energy (and oxygen) demands are highest^29^. While in previous studies we had attributed the baseline level of [^64^Cu][Cu(ATSM)] cardiac retention in “normoxically-perfused” isolated hearts to be due to non-specific retention in cell membranes (due to their lipophilicity), our data suggest that some of this retention may represent a small fraction of hypoxic cells within a crystalloid-perfused heart, which become normoxic once when the heart is mechanically unloaded. We have recently shown by ^31^P NMR that mechanically uncoupling isolated hearts with blebbistatin elevates both phosphocreatine levels and ATP content in “normoxic” crystalloid buffer-perfused isolated hearts which would be consistent with this^30^.

We exploited the ammonium prepulse approach to specifically induce intracellular acidosis, as opposed to perfusing with acidotic buffers, which would not necessarily translate to intracellular acidosis. This also allowed us to exclude the possibility of protonation of the complex by acidotic buffer before it reached the heart in our study. The ammonium prepulse approach coupled with NHE inhibition is an established means of inducing and maintaining intracellular acidosis in both basic cardiac research and other applications^31,32^; similar, but less severe intracellular acidosis of pH 6.7 has previously been demonstrated with a similar protocol in isolated perfused ferret hearts using the NHE inhibitor 5-(N-ethyl-N-isopropyl)amiloride (EIPA)^33^, while similar effects on contractile function to those we report here have previously been demonstrated with NH_4_Cl infusion and NHE-1 inhibitor cariporide^34^. While NHE inhibition is associated with a loss of Ca^2+^ uptake via NCX, acidosis-mediated loss of contractile function is not due to the decrease in Ca^2+^ concentration, but to a decrease in contractile protein responsiveness to Ca^2+ ^^35^. We demonstrate that zoniporide treatment alone had no effect on Copper-64 retention during normoxia or hypoxia compared to vehicle control, which confirms that there was no interaction between zoniporide and [^64^Cu][Cu(ATSM)] which may have affected myocardial uptake or dissociation.

## Conclusion

Radiocopper bis(thiosemicarbazone) complexes represent a versatile family of hypoxia imaging agents with a range of hypoxia selectivities for a variety of applications in both cardiology and oncology. To optimize the diagnostic and prognostic insight gained from the PET images with these complexes, it is essential to understand the nature of their tissue uptake and retention. Here, we demonstrate that their hypoxia-dependent tissue retention is not dependent upon intracellular acidosis (nor indeed directly affected by it) and confirm their specificity to changes in intracellular oxygen saturation.

## Materials and Methods

### Reagents and gas mixtures

All reagents were purchased from Sigma Aldrich (Poole, Dorset, UK) unless otherwise stated. All gas mixtures were purchased from BOC, UK. Specialist gas mixtures were certified by the manufacturer.

### [^64^Cu][Cu(ATSM)] production

Copper-64 was provided by the PET Imaging Centre, St. Thomas’ Hospital, London. ATSM was labeled with Copper-64 as described by Handley *et al*.^36^.

### Animals

Male Wistar rats (250 to 300 g: Charles River) with *ad libitum* access to food and water were used throughout. All experimental procedures were approved by King’s College London’s local Animal Care and Ethics Committee, and carried out in accordance with Home Office regulations as detailed in the Guidance on the Operation of Animals (Scientific Procedures) Act 1986.

### Heart perfusion protocol

**Figure 7:**
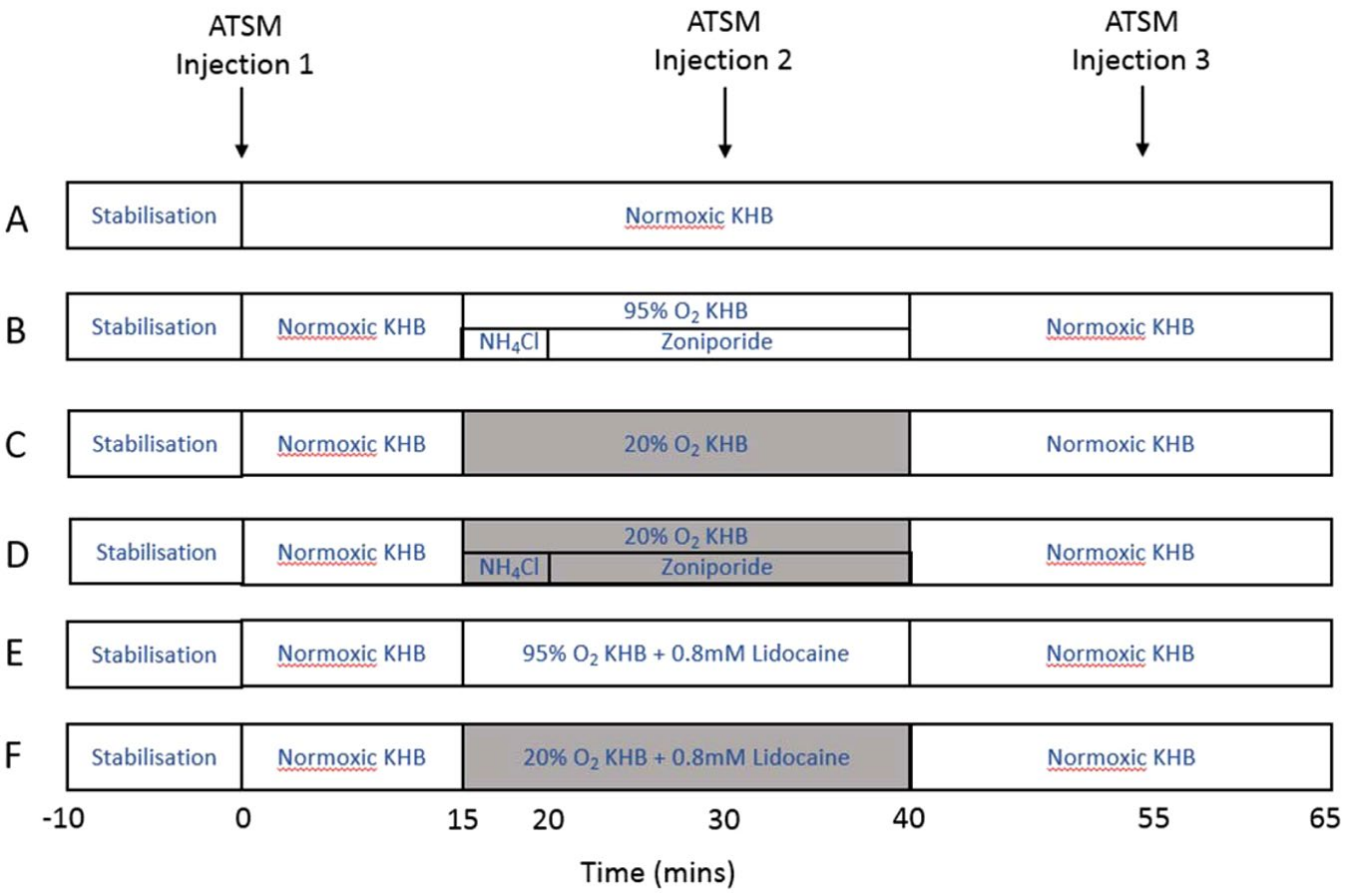
Perfusion protocols for hearts from all treatment groups. (**A**) Normoxic control, (**B**) Normoxia + acidosis (**C**) Hypoxic control (**D**) Hypoxia + acidosis (**E**) Normoxia + lidocaine infusion (**F)** Hypoxia + lidocaine infusion.

Rats were anesthetised with sodium pentobarbitone and heparinised (200 IU intraperitoneal). Hearts were excised and placed immediately in Krebs-Henseleit Buffer (KHB) at 4 °C, comprising NaCl (118 mmol/L), KCl (5.9 mmol/L), MgSO_4_ (1.16 mmol/L), NaHCO_3_ (25 mmol/L), NaEDTA (0.48 mmol/L), glucose (11.1 mmol/L) and CaCl_2_ (2.2 mmol/L) prior to Langendorff perfusion at a constant rate of 14 mL/min with KHB gassed with 95%O_2_/5%CO_2_ at 37 °C, as we have described previously^12,37^. Hypoxia was induced by switching to KHB equilibrated with 20%O_2_/75%N_2_/5%CO_2_. Buffer oxygen saturation was monitored throughout each experiment by an OxyLite™ fluorescent oxygen probe (Oxford Optronix Ltd., Oxfordshire, UK) inserted into the arterial perfusion line. Coronary perfusion pressure was monitored via a pressure transducer mounted in the arterial line. Cardiac contractile function was monitored via a pressure transducer connected to a latex balloon inserted into the left ventricle, inflated to an end-diastolic pressure of 4 to 9 mmHg. All hearts were perfused with normoxic KHB for a stabilization period of 10 min to ensure contractile function exclusion criteria were met before continuing each experiment. Hearts were then perfused for a further 45 min according to the protocol schematics in Fig. 7. In each experiment, three boluses of [^64^Cu][Cu(ATSM)] (2 MBq in 100 μL KHB) were injected into the arterial perfusion line at the end of the stabilization period (to obtain a baseline measurement), 20 minutes after the induction of each intervention, and 15 minutes after the cessation of each intervention. An ammonium pre-pulse technique was employed to induce intracellular acidosis^38^, which was maintained by infusion of the sodium hydrogen exchanger (NHE-1) inhibitor zoniporide^39^ in bicarbonate free buffer (to limit buffering capacity). Hypoxia was induced by perfusing hearts with KHB gassed with 20% O_2_. Since tissue acidosis inhibits cardiac contractility, which would potentially have an oxygen-salving effect which may itself affect hypoxia tracer pharmacokinetics, we perfused further groups of hearts with 0.8 mM lidocaine to inhibit cardiac contractility to establish the effect of tissue hypocontractility on tracer pharmacokinetics independent of intracellular pH.

### ^31^P MR Spectroscopic Analysis

Changes in cardiac energetics and intracellular pH were monitored in real time in parallel groups of hearts using ^31^P NMR spectroscopy. Hearts were cannulated and perfused with KHB in Langendorff mode and inserted into a 15 mm glass MR tube, which was inserted into a custom-built MR spectroscopy probe, as previously described^40^. ^31^P NMR spectra were acquired on an Bruker Avance III 9.4 T spectrometer using a 15 mm ^31^P/^1^H birdcage coil^41^. Shimming was performed on the ^1^H line shape of water (full width at half maximum <20 Hz). ^31^P spectra were acquired with a pulse-acquire sequence using a 60° flip angle, a repetition time of 3.8 s, and 64 scans (4 min per spectrum). The peak area of each metabolite was normalized to that of phosphocreatine during normoxia.

### Lactate measurement

0.5 mL of coronary effluent was collected at regular intervals during perfusion and analyzed for lactate content to identify the onset of anaerobic glycolysis using a 2300 STAT Plus lactate analyzer (YSI Ltd) as described previously^37^.

### Radiometric measurement

Cardiac radiotracer injection, retention and washout was monitored throughout using three orthogonally-arranged lead-collimated Na/I γ-radiation detectors (Raytest Isotopenmessgeräte GmbH, Straubenhardt, Germany) measuring Copper-64 activity at the input (arterial) perfusion line, the heart and the output perfusion line respectively. The detectors were connected to a Gina Star™ data acquisition system (Raytest Isotopenmessgeräte GmbH) as previously described^15^. Data were normalized to the maximum peak counts after each injection and corrected for decay and cardiac background activity 30 seconds prior to each injection.

### Statistical analysis

All data are presented as mean ± standard deviation. Statistical significance was calculated using a one-way ANOVA followed by Bonferroni *post hoc* test, or Dunnett test when multiple comparisons were made to a control group, using GraphPad Prism (GraphPad software Inc., San Diego, CA, USA).

## Data Availability

The datasets generated during and/or analysed during the current study are available from the corresponding author on reasonable request.
